# Neck Circumference as a Surrogate Marker of Upper-Body Adiposity: Associations with Anthropometric, Biochemical and Inflammatory Cytokines in Mexican Adults with Obesity

**DOI:** 10.3390/nu18091366

**Published:** 2026-04-26

**Authors:** Samantha Desireé Reyes-Perez, Evelyn Valencia-Sosa, Roberto Rodriguez-Echevarria, Joel Torres-Vanegas, Diego Cambron-Mora, Erika Martínez-López

**Affiliations:** Instituto de Nutrigenética y Nutrigenómica Traslacional, Departamento de Biología Molecular y Genómica, Centro Universitario de Ciencias de la Salud, Universidad de Guadalajara, Guadalajara 44340, Mexico; samantha.reyes@academicos.udg.mx (S.D.R.-P.); evelyn.vs@tec.mx (E.V.-S.); roberto.rodriguez@academico.udg.mx (R.R.-E.);

**Keywords:** neck circumference, adiposity, obesity, metabolic alterations, inflammation, cytokines, Interleukin-10

## Abstract

**Background:** Neck circumference (NC) is a simple anthropometric index of upper-body subcutaneous adiposity. Its relationship with inflammatory cytokines, particularly the anti-inflammatory cytokine IL-10, remains poorly explored, especially in Latin-American populations. **Methods:** This cross-sectional study analyzed data from 80 Mexican adults with obesity (40 women and 40 men). NC, waist circumference (WC), and body mass index (BMI) were measured according to standardized ISAK and NHANES protocols. Fasting glucose, insulin, lipid profile, and HbA1c were evaluated. Finally, serum TNF-α, IL-6, IL-18, IL-10, and hs-CRP were quantified from a subsample. Correlation coefficients and sex-stratified multiple linear regression models adjusted for age and BMI were performed. **Results:** In the overall sample, neck circumference (NC) was positively associated with triglycerides, VLDL-C, insulin, and HbA1c, and inversely associated with HDL-C. Sex-stratified analyses revealed marked differences. In women, NC showed strong and independent associations with triglycerides, VLDL-C, IL-10, and the IL-10/TNF-α ratio, whereas these associations were not observed in men. Similarly, waist circumference was independently associated with IL-10, and BMI was associated with hs-CRP. **Conclusions:** NC may serve as a useful surrogate marker of upper-body adiposity and is independently associated with triglycerides, VLDL-C, IL-10, and the IL-10/TNF-α ratio exclusively in women with obesity. These exploratory findings highlight sex-specific differences in the adiposity–inflammation axis and underscore the need for larger longitudinal studies and sex-tailored approaches in obesity research.

## 1. Introduction

Obesity constitutes a major global public health challenge, with its prevalence increasing substantially in recent decades and conferring a markedly elevated risk of metabolic disorders, including insulin resistance, dyslipidemia, type 2 diabetes mellitus, and cardiovascular disease [1,2]. Beyond total adiposity, the regional distribution of adipose tissue is a determinant of cardiometabolic complications; particularly upper-body fat accumulation has been consistently associated with a more adverse metabolic and inflammatory profile [2].

In clinical practice, body mass index (BMI) remains the most widely used anthropometric index to classify nutritional status. However, BMI has well-documented limitations: it fails to differentiate fat mass from lean mass and provides no information on fat distribution [3,4]. Waist circumference (WC) partially addresses the latter but is subject to measurement variability related to posture, respiration movements, and postprandial state [5,6]. In contrast, neck circumference (NC) offers several distinct advantages as an alternative marker of upper-body subcutaneous adiposity [7,8,9]. It is simple, inexpensive, highly reproducible, stable throughout the day, and does not require undressing or specialized equipment, making it particularly suitable for large-scale epidemiological studies and routine clinical screening [7,10,11]. Moreover, NC correlates strongly with visceral adipose tissue and cardiometabolic risk factors independently of BMI and WC in diverse populations, including Latin-American adults [12]. In Mexican populations, NC has also been associated with traditional anthropometric indicators and components of metabolic syndrome, including dyslipidemia and hyperglycemia, and population-specific cutoff points have been proposed. However, evidence in Mexican adults with obesity, particularly regarding inflammatory biomarkers, remains limited [13].

Cervical adipose tissue is metabolically active and releases free fatty acids that can promote insulin resistance and dyslipidemia [14,15,16]. Consequently, elevated NC has been linked to key cardiometabolic markers such as elevated triglycerides, reduced high-density lipoprotein cholesterol, hyperinsulinemia, and hyperglycemia [14]. Nevertheless, these associations remain inconsistent in individuals with established obesity, where alterations in body composition may modulate the relationship [8].

In addition, NC may serve as an indicator of a pro-inflammatory state. Upper-body subcutaneous fat depots contribute to the systemic release of pro-inflammatory adipokines and cytokines, including Tumor necrosis factor-alpha (TNF-α), Interleukin-6 (IL-6), and Interleukin-18 (IL-18) [17]. Conversely, the anti-inflammatory cytokine Interleukin-10 (IL-10) has received far less attention in this context. Although a limited number of studies have examined the association between NC and inflammatory markers [18], evidence assessing its relationship with both pro- and anti-inflammatory cytokines, particularly Interleukin-10 (IL-10), in adults with established obesity remains scarce. This gap is especially relevant in Mexican populations from Western Mexico, where genetic, dietary, and environmental factors may modulate the adiposity–inflammation axis.

The aim of the present study is to evaluate the association of NC with anthropometric, metabolic, and inflammatory markers, and to compare its performance with traditional anthropometric indicators such as WC and BMI.

## 2. Materials and Methods

### 2.1. Study Design and Setting

This analytical cross-sectional study analyzed baseline data from participants enrolled in the EPICO project (Estudio para la Pro-resolución de la Inflamación Crónica en Obesidad) [19]. For the present analysis, only baseline data from participants were considered, therefore a non-probability sampling method was employed. The study was conducted at the Instituto de Nutrigenética y Nutrigenómica Traslacional (INNUGET), Universidad de Guadalajara, Mexico.

### 2.2. Participants

Participants were Mexican adults aged 25–59 years with obesity, defined as a body mass index (BMI) ≥30 kg/m^2^ or the presence of abdominal obesity according to ATP III criteria (WC > 88 cm in women and >102 cm in men). A total of 80 participants (40 men and 40 women) were included in the study. Anthropometric and metabolic measurements were obtained for the entire sample.

Exclusion criteria comprised a prior diagnosis of chronic cardiovascular, metabolic, endocrine, neoplastic, or autoimmune diseases; pregnancy or breastfeeding; use of anti-inflammatory, lipid-lowering, or hypoglycemic medications; consumption of fatty acid supplements within the previous three months; history of gastrointestinal surgery (including cholecystectomy); alcohol abuse; or weight loss exceeding 10% of body weight in the prior three months. Additional inclusion criteria stipulated that subjects had not attempted any substantial lifestyle modifications regarding physical activity or dietary habits (e.g., calorie restriction) to maintain comparable baseline conditions across participants. Participants were recruited from the Guadalajara metropolitan area through social media and open calls, with eligibility assessed via virtual screening.

### 2.3. Variables and Instruments

Anthropometric, metabolic, and inflammatory variables were assessed using standardized and validated methods.

Anthropometric variables included body weight, height, body mass index (BMI), neck circumference (NC), waist circumference (WC), hip circumference, and body composition parameters (body fat percentage and skeletal muscle mass). All anthropometric measurements were performed by trained personnel following standardized protocols of the International Society for the Advancement of Kinanthropometry (ISAK) [20] and the National Health and Nutrition Examination Survey (NHANES). Measurements were obtained after an 8–12 h overnight fast, with participants barefoot and wearing light clothing. Equipment was calibrated according to the manufacturer’s specifications prior to data collection to ensure accuracy and reproducibility. Height was measured using a stadiometer (SECA, Hamburg, Germany, precision of 1 mm) with the head positioned in the Frankfurt plane. BMI was calculated as weight in kilograms divided by height in meters squared (kg/m^2^). Neck circumference (NC) was measured using a non-elastic metal tape (Lufkin model W606, precision 0.1 cm (Apex Tool Group, Sparks, MD, USA)) positioned horizontally at the mid-neck level, just below the laryngeal prominence and perpendicular to the long axis of the neck. Waist and hip circumferences were measured according to ISAK guidelines. Body composition, including body fat percentage, was assessed by tetrapolar bioelectrical impedance analysis using an InBody 570 device (Biospace Co., Ltd., Seoul, Republic of Korea) under standardized conditions.

Metabolic variables included fasting glucose, total cholesterol, high-density lipoprotein cholesterol (HDL-C), triglycerides, low-density lipoprotein cholesterol (LDL-C), very low-density lipoprotein cholesterol (VLDL-C), insulin, and glycated hemoglobin (HbA1c). Fasting blood samples were collected via the vacutainer system after 8–12 h of fasting. Samples were centrifuged at 4 °C for 15 min at 3500 RPM to obtain serum, which was stored at −80 °C until analysis. Biochemical analyses were performed using standardized and validated laboratory methods following the manufacturers’ instructions. Serum glucose, total cholesterol, high-density lipoprotein cholesterol (HDL-C), and triglycerides were measured by dry chemistry using a Vitros 350 Analyzer (Ortho-Clinical Diagnostics, Johnson & Johnson Services Inc., Rochester, NY, USA). Low-density lipoprotein cholesterol (LDL-C) was calculated using the Friedewald formula when triglyceride levels were <400 mg/dL, and very low-density lipoprotein cholesterol (VLDL-C) was estimated as triglycerides divided by 5. Insulin was quantified by chemiluminescent immunoassay using a Liaison analyzer (DiaSorin S.p.A., Saluggia, Italy). Glycated hemoglobin (HbA1c) was determined by a certified external laboratory using a chemiluminescent method.

Serum inflammatory markers (TNF-α, IL-6, IL-10, and IL-18) were quantified because of their well-documented involvement in obesity-associated low-grade chronic inflammation and in the progression of metabolic dysfunction. These markers were measured in duplicate using ProQuantum High Sensitivity Immunoassay kits (Invitrogen by Thermo Fisher Scientific, Carlsbad, CA, USA) according to the manufacturers’ instructions. High-sensitivity C-reactive protein (hs-CRP) was measured by immunofluorescence using the Getein 1100 (Getein Biotech, Nanjing, China) equipment. Inflammatory markers were assessed only in a subsample of 55 participants (24 women and 31 men) who successfully completed the 8-week EPICO randomized clinical trial. Cytokine determinations were performed exclusively in these individuals, as participants who withdrew from the trial did not undergo cytokine analysis. Consequently, only baseline data from this subsample were included in the present cross-sectional analysis. In addition, the IL-10/TNF-α ratio was calculated for each participant by dividing the serum concentration of IL-10 by the serum concentration of TNF-α (both expressed in pg/mL).

### 2.4. Statistical Analysis

Statistical analyses were performed using IBM SPSS Statistics, version 25.0 (IBM Corp., Armonk, NY, USA). Normality was assessed using the Kolmogorov–Smirnov test for the overall sample and the Shapiro–Wilk test for sex-stratified analyses. All continuous variables are presented as mean ± standard deviation. Differences between sexes were evaluated using the independent samples *t*-test or the Mann–Whitney U test, as appropriate according to data distribution. Associations between NC, WC, and BMI with anthropometric, metabolic, and inflammatory variables were evaluated using Pearson’s or Spearman’s correlation coefficients, as appropriate according to the results of the Shapiro–Wilk test. Multiple linear regression analyses using the enter method were conducted to examine the independent associations of NC, WC, and BMI with metabolic and inflammatory markers. Models for NC and WC were adjusted for age, sex, and BMI, while models for BMI were adjusted for age and sex only. The effect sizes were reported as R^2^ for each model and as standardized beta (β) regression coefficients for individual predictors. Multicollinearity diagnostics were performed for all regression models. Variance inflation factors (VIF) were calculated, and a threshold of <2.0 was considered optimal to confirm the absence of multicollinearity issues and ensure reliable interpretation of independent associations. Given the exploratory nature of this study and the relatively small sample size for cytokine analyses, no correction for multiple testing was applied. Therefore, all reported *p*-values should be considered hypothesis-generating. In all statistical tests, a two-tailed *p*-value < 0.05 was considered statistically significant.

### 2.5. Ethical Considerations

The study protocol was approved by the Institutional Ethics, Research, and Biosafety Committee of the Centro Universitario de Ciencias de la Salud, Universidad de Guadalajara (approval code CI-03221) and registered at ClinicalTrials.gov (NCT05068557). The procedures were conducted in accordance with the Declaration of Helsinki (2013). Written informed consent was obtained from all participants prior to any data collection, and confidentiality of personal information was strictly maintained.

## 3. Results

A total of 80 Mexican adults with obesity (40 women and 40 men) were included in the analysis of anthropometric and metabolic variables. Inflammatory markers were quantified in a subsample of 55 participants (24 women and 31 men) who completed the 8-week EPICO randomized clinical trial. To evaluate potential selection bias, baseline anthropometric and metabolic characteristics were compared between participants in whom inflammatory markers were measured (completers, *n* = 55) and those in whom they were not measured (non-completers, *n* = 25). No statistically significant differences (*p* < 0.05) were observed between the two groups in most key variables examined (including weight, height, NC, WC, BMI, BFM and SMM). However, the age of the individuals in the subsample was higher (39.0 ± 9.8 vs. 34.1 ± 8.4 years, *p* = 0.018) according to the Mann–Whitney U test.

Baseline characteristics of the study population, stratified by sex, are presented in Table 1. Men showed higher weight and height than women and exhibited higher skeletal muscle mass and WC (*p* < 0.001). In contrast, no significant sex differences were observed in age, BMI, or body fat mass (BFM). Regarding metabolic parameters, men displayed significantly higher levels of triglycerides and VLDL-C, as well as lower HDL-C levels compared with women (*p* < 0.001). No statistically significant sex differences were found in fasting glucose, HbA1c, insulin, or any of the measured inflammatory markers, including hs-CRP, TNF-α, IL-6, IL-10, IL-18, and the IL-10/TNF-α ratio.

Correlation analyses were first conducted in the overall sample to provide a general overview of the relationships between variables. NC, BMI, and WC showed distinct patterns of association with anthropometric and metabolic variables in the overall sample (Table 2). NC was strongly positively correlated with body weight (r = 0.68, *p* < 0.001), skeletal muscle mass (r = 0.79, *p* < 0.001), and WC (r = 0.84, *p* < 0.001). It also exhibited significant positive correlations with triglycerides (r = 0.43, *p* < 0.001), VLDL-C (r = 0.43, *p* < 0.001), insulin (r = 0.41, *p* = 0.004), and HbA1c (r = 0.24, *p* = 0.03), as well as a moderate inverse correlation with HDL-C (r = −0.44, *p* < 0.001). WC demonstrated the strongest correlation with body weight (r = 0.83, *p* < 0.001) and was significantly associated with triglycerides (r = 0.30, *p* = 0.006), VLDL-C (r = 0.30, *p* = 0.006), HDL-C (r = −0.40, *p* < 0.001), and HbA1c (r = 0.23, *p* = 0.039). BMI showed particularly strong associations with body weight (r = 0.69, *p* < 0.001), body fat mass (r = 0.87, *p* < 0.001) and was positively correlated with glucose (r = 0.28, *p* = 0.013) and hs-CRP (r = 0.578, *p* < 0.001). No significant correlations were observed between any of the three anthropometric indices and the rest of the measured inflammatory markers in the overall sample.

Subsequently, sex-stratified correlations were conducted to assess sex-specific patterns, given known biological differences in fat distribution, hormonal regulation, and inflammatory responses between men and women. Sex-stratified bivariate correlation analyses showed important differences between women and men (Table 3). In women, NC showed moderate to strong positive correlations with weight (r = 0.37, *p* = 0.019), BMI (r = 0.48, *p* = 0.002), and WC (r = 0.70, *p* < 0.001). NC was also significantly correlated with triglycerides (r = 0.63, *p* < 0.001), VLDL-C (r = 0.64, *p* < 0.001), IL-10 (r = 0.77, *p* < 0.001), and the IL-10/TNF-α ratio (r = 0.677, *p* = 0.008). WC demonstrated moderate to strong positive correlations with weight (r = 0.58, *p* < 0.001), BMI (r = 0.48, *p* = 0.002), triglycerides (r = 0.36, *p* = 0.020), VLDL-C (r = 0.36, *p* = 0.021) and HbA1c (r = 0.37, *p* = 0.018). BMI was positively correlated with weight (r = 0.69, *p* < 0.001), BFM (r = 0.81, *p* < 0.001) WC (r = 0.66, *p* < 0.001), glucose (r = 0.37, *p* = 0.02), and hs-CRP (r = 0.62, *p* < 0.008).

In men, the pattern differed considerably. NC was positively correlated with weight (r = 0.49, *p* = 0.001), BMI (r = 0.52, *p* = 0.001), and WC (r = 0.65, *p* = 0.017), but showed inverse associations with total cholesterol (r = −0.49, *p* = 0.001), HDL-C (r = −0.37, *p* = 0.020), and LDL-C (r = −0.40, *p* = 0.010). WC exhibited strong positive correlations with weight (r = 0.84, *p* < 0.001), and BMI (r = 0.82, *p* < 0.001). BMI showed strong positive correlations with weight (r = 0.90, *p* = 0.001) and WC (r = 0.82, *p* < 0.001), but negative correlations with LDL-C (r = −0.33, *p* < 0.034).

To further assess the independent associations of the three anthropometric indices, sex-stratified multiple linear regression models were performed (Table 4). This analytical approach was selected to account for sex as a potential effect modifier, thereby enabling a more accurate estimation of associations within each group and minimizing the risk of masking sex-specific effects. Models for NC and WC were adjusted for age and BMI to account for overall adiposity and to determine whether these regional anthropometric measures are independently associated with metabolic and inflammatory outcomes beyond general body mass, whereas models for BMI were adjusted for age and sex only. Although these indices are conceptually related, they capture distinct aspects of fat distribution.

In women, NC emerged as a strong and independent marker associated with several metabolic and inflammatory markers. It was significantly associated with higher triglycerides (β = 0.780, R^2^ = 0.506, *p* < 0.001), higher VLDL-C (β = 0.782, R^2^ = 0.505, *p* < 0.001), higher IL-10 *(*β = 0.890, R^2^ = 0.569, *p* = 0.001) and a higher IL-10/TNF-α ratio (β = 0.861, R^2^ = 0.446, *p* = 0.005). Similarly, in some variables, WC showed a significant independent association with higher triglycerides (β = 0.560, R^2^ = 0.238, *p* = 0.005), higher VLDL-C (β = 0.554, R^2^ = 0.147, *p* = 0.005) IL-10 (β = 0.902, R^2^ = 0.356, *p* = 0.011). In contrast, BMI displayed an independent association with higher hs-CRP (β = 0.583, R^2^ = 0.307, *p* = 0.023).

In men, the pattern was markedly different. NC was inversely and independently associated with total cholesterol (β = −0.429, R^2^ = 0.340, *p* = 0.013), HDL-C (β = −0.465, R^2^ = 0.147, *p* = 0.018), and LDL-C (β = −0.439, R^2^ = 0.187, *p* = 0.021). WC showed no significant independent associations with any variable. BMI was inversely associated with total cholesterol (β = −0.499, R^2^ = 0.236, *p* = 0.001) and LDL-C (β = −0.322, R^2^ = 0.081, *p* = 0.044). No significant independent associations were observed between any of the three anthropometric indices and IL-10 or the IL-10/TNF-α ratio in men.

Multicollinearity diagnostics were performed for all sex-stratified multiple linear regression models. Variance inflation factors (VIF) for NC ranged from 1.34 to 1.60, for WC from 1.92 to 1.96, and for BMI from 1.01 to 1.04. All VIF values were well below 2.0 in both women and men, indicating the absence of multicollinearity issues and ensuring reliable interpretation of the independent associations of the three anthropometric indices.

## 4. Discussion

This cross-sectional analysis showed that NC is independently associated with both cardiometabolic and anti-inflammatory markers. NC exhibited consistent positive associations with key anthropometric measures (body weight, body fat mass, skeletal muscle mass, and WC) along with several metabolic parameters, including triglycerides and VLDL-C. These associations remained significant in sex-stratified models after adjustment for age and BMI. Most notably, NC demonstrated strong independent positive associations with the anti-inflammatory cytokine IL-10 and the IL-10/TNF-α ratio exclusively in women. Noteworthy, models for NC and WC were adjusted for BMI because these indices reflect regional adiposity (upper-body subcutaneous and central, respectively), whereas BMI captures overall adiposity. This adjustment was performed to evaluate the independent contribution of regional fat distribution beyond total body size, despite the conceptual overlap among the three anthropometric measures. Although residual collinearity was statistically negligible, we acknowledge that over-adjustment remains a theoretical possibility in highly correlated anthropometric variables.

The associations between NC and classical cardiometabolic risk factors are consistent with the existing literature. Multiple studies and meta-analyses have described NC as a simple, reproducible surrogate marker of upper-body subcutaneous adiposity that correlates with altered lipid profiles, insulin resistance, and cardiovascular risk, often independently of BMI and WC [9,12,21]. Although NC demonstrated stronger independent associations with several cardiometabolic and inflammatory markers, it is important to remark that WC and BMI remain highly valuable anthropometric indices with large-scale applications. These conventional measures provide complementary information to NC, each capturing distinct aspects of adiposity and cardiometabolic risk.

In the cardiometabolic domain, NC and WC consistently demonstrated higher correlations with key metabolic parameters compared to BMI. These findings suggest that NC and WC reflect metabolically relevant aspects of upper-body adiposity that are not fully accounted for by BMI alone. In contrast, only BMI displayed significant positive correlations with blood glucose and hs-CRP. Data from Latin-American cohorts on NC and cardiometabolic risk remain limited, with some studies focusing only the pediatric population, leaving important questions unanswered regarding implications in adults.

Regarding inflammatory markers, the differences were even more pronounced. NC exhibited strong positive correlations with IL-10 and the IL-10/TNF-α ratio exclusively in women. Noteworthy, this correlation analysis was exploratory, as stated in the Section 2. Second, circulating levels of IL-10 are known to fluctuate according to metabolic state and should not be considered a direct reflection of overall anti-inflammatory capacity. In addition, the present study did not account for potentially relevant confounding factors, such as hormonal status, physical activity, or dietary patterns, which may influence inflammatory responses. Therefore, any interpretation suggesting sex-specific biological mechanisms remains speculative and warrants confirmation in future studies.

Evidence specifically linking NC to circulating cytokines, particularly the anti-inflammatory cytokine IL-10, remains limited. Most prior research has focused on pro-inflammatory markers such as CRP, E-selectin, plasminogen activator inhibitor 1 (PAI-1), TNF-α and Interleukin-6 (IL-6) [18,22] leading to interesting results linking NC with E-selectin and PAI-1. Similarly in children, other articles have found relevant data mostly on adipokines such as leptin and adiponectin [23,24]. The scarcity of studies examining IL-10 in relation to regional adiposity indices such as NC is therefore notable, especially because IL-10 dysregulation has been implicated in the progression from metabolically healthy to metabolically unhealthy obesity [25,26]. However, IL-10 is a highly context-dependent cytokine whose biological effects vary according to the inflammatory milieu. In the setting of obesity, elevated circulating IL-10 levels are frequently interpreted as a compensatory anti-inflammatory response aimed at restraining chronic low-grade inflammation, rather than reflecting an overall improvement in inflammatory status [27,28].

The findings observed suggest that associations with IL-10 and the IL-10/TNF-α ratio in women may reflect sex-related patterns in adipose tissue distribution, hormonal and immune factors. [1,26]. In the context of obesity, low circulating IL-10 levels have been linked to greater metabolic dysfunction, while preserved or elevated IL-10 production may exert a compensatory role against the progression of low-grade chronic inflammation [25,27]. Consistent with this, Esposito et al. [27] demonstrated that women with obesity had higher circulating IL-10 than subjects without obesity, yet those with the metabolic syndrome exhibited significantly lower IL-10 levels. More recently, Subramanian et al. [25] reported a clear sexual dimorphism in human adipose tissue, showing that women with obesity and type 2 diabetes mellitus (T2D) displayed markedly increased IL-10 expression in subcutaneous white adipose tissue, along with a higher IL-10 secretion, which was also reflected in circulating levels of this cytokine compared to women without obesity. The authors proposed that this sex-specific increase in IL-10 may represent a protective mechanism against obesity-associated chronic inflammation in women. Interestingly, estrogens are known to enhance IL-10 production and to modulate adipocyte inflammatory responses, while upper-body subcutaneous fat depots in women may exhibit distinct secretory profiles compared with those in men [25,29]. Given this established sexual dimorphism in adipose tissue biology and inflammatory regulation, we performed both overall and sex-stratified correlation analyses to provide a comprehensive overview of the associations while exploring potential effect modification by sex. Accordingly, multiple linear regression models were conducted exclusively on a sex-stratified basis to avoid masking sex-specific effects and to appropriately evaluate the independent contribution of NC within each sex.

Furthermore, the IL-10/TNF-α ratio has been studied in multiple pathologic scenarios. Specifically, in the context of cardiometabolic alterations, this ratio is considered as a useful indicator of the balance between pro- and anti-inflammatory signals. In obesity and the metabolic syndrome, a higher IL-10/TNF-α ratio has been interpreted as a compensatory anti-inflammatory response aimed at counteracting chronic low-grade inflammation driven by excess adipose tissue [29,30]. Sinatora V. et al. [29] proposed that the IL-10/TNF-α ratio could identify individuals with metabolic syndrome according to the NCEP ATP III criteria.

From a physiological and molecular perspective, cervical adipose tissue is metabolically active and releases free fatty acids and adipokines that can influence systemic inflammation [14]. The positive associations between NC and WC with IL-10 in women may add some evidence of the inflammatory events related to upper-body adiposity [25,27]. This finding is especially relevant in Mexican populations, where genetic, dietary, and environmental factors may modulate the adiposity–inflammation axis differently than in other ethnic groups [26,31]. The absence of this association in men could contribute to their generally higher cardiometabolic risk at similar levels of obesity.

From a clinical and public health perspective, the present findings suggest that NC may represent a practical and accessible anthropometric marker associated with increased cardiometabolic risk. Its independent associations with metabolic parameters and its relationship with anti-inflammatory markers in women indicate that NC could provide complementary information beyond traditional indices such as WC and BMI. However, given the cross-sectional design, these findings should be interpreted as associations rather than evidence of predictive or diagnostic utility.

The present study has several strengths. It derives from a well-characterized randomized clinical trial with standardized anthropometric and laboratory protocols and focuses on an understudied Latin-American population. In addition, it is important to remark that NC stands out as a simple, inexpensive, stable, and highly reproducible measurement that has extensively been proposed to be included in large-scale epidemiological studies and routine clinical screening. This measurement may be particularly useful in individuals with obesity, in whom traditional anthropometric assessments such as WC can be technically challenging due to body size, positioning difficulties, and the need for partial undressing, which may be perceived as invasive or uncomfortable. Moreover, the use of both global and sex-stratified analyses provides additional insight into potential effect modifiers. Nevertheless, several limitations should be acknowledged. An additional limitation relates to the subsample in which inflammatory cytokines were measured. Most demographic, anthropometric, and metabolic variables were comparable between the full cohort and the subsample, although the latter was statistically significantly older, which may indicate a potential source of selection bias. Therefore, cytokine-related findings should be interpreted with caution. Although multicollinearity was not detected in any model (VIF < 2.0), the relatively small sample size for cytokine analyses (*n* = 55; 24 women and 31 men) limits statistical power, particularly in sex-stratified regression models, and raises the possibility of overfitting in models with relatively high adjusted R^2^ values. Consequently, findings, particularly those related to IL-10 and IL10/TNF-α ratio should be interpreted as preliminary and exploratory. In addition, the large number of statistical tests performed, including multiple correlations and regression analyses, increases the risk of type I error, further underscoring the need for cautious interpretation of the results, especially those involving inflammatory markers. The cross-sectional design precludes causal inferences, and residual confounding from unmeasured factors (e.g., physical activity, dietary patterns, or hormonal status) cannot be entirely ruled out. Finally, larger prospective studies, including normal-weight individuals, are therefore warranted to confirm these sex-specific patterns and to further elucidate the underlying mechanisms.

## 5. Conclusions

Our results suggest that NC may serve as a useful surrogate marker of upper-body adiposity. It is independently associated with serum triglycerides and VLDL-C, as well as with IL-10 and the IL-10/TNF-α ratio, exclusively in women with obesity. Given the exploratory nature of these findings and the relatively small subsample for cytokine analyses, confirmation in larger longitudinal cohorts is essential to establish their clinical relevance. The observed sex-specific differences further highlight the importance of considering sex as a biological variable and support the development of sex-tailored strategies in the management of obesity-related inflammation.

## Figures and Tables

**Table 1:** Participant characteristics by sex.

Variable		Women		Men		*p*
		Mean	SD	Mean	SD	
Anthropometric	Age (years)	36.7	9.5	35.9	9.3	0.718
	Weight (kg)	90.3	10.9	108.2	15.1	<0.001
	Height (cm)	162.4	7.1	174.9	5.5	<0.001
	SMM (kg)	26.3	3.0	36.1	3.7	<0.001
	BFM (kg)	42.5	7.3	44.2	11.1	0.769
	BMI (kg/m^2^)	34.2	3.1	35.3	4.3	0.288
	WC (cm)	96.7	7.6	112.1	9.3	<0.001
	NC (cm)	36.3	2.3	42.9	2.6	<0.001
Metabolic	CHOL (mg/dL)	165.3	29.1	171.7	28.4	0.327
	Triglycerides (mg/dL)	148.7	62.2	207.1	81.5	0.001
	HDL-C (mg/dL)	44.5	12.1	36.2	9.1	0.001
	LDL-C (mg/dL)	90.7	20.6	94.2	24.8	0.500
	VLDL-C (mg/dL)	29.8	12.5	41.5	16.3	0.001
	Glucose (mg/dL)	91.6	7.6	99.0	24.2	0.289
	HbA1c (%)	5.3	0.4	5.5	0.6	0.294
	Insulin (UI)	17.5	11.1	20.6	10.1	0.110
Inflammatory	hs-CRP (mg/L)	3.7	2.2	3.8	1.9	0.951
	TNF-α (pg/mL)	0.9	1.0	0.8	0.8	0.920
	IL-6 (pg/mL)	4.3	3.6	4.0	4.3	0.475
	IL-10 (pg/mL)	7.1	5.6	6.3	3.0	0.734
	IL-18 (pg/mL)	128.7	32.6	130.2	38.9	0.927
	IL-10/TNF-α ratio	18.3	39.0	13.0	11.3	0.529

Comparisons between sexes were performed using the independent samples *t*-test (anthropometric and metabolic variables) or Mann–Whitney U test (inflammatory variables), as appropriate, according to normality tests. Anthropometric and metabolic variables were analyzed in the overall sample (*n* = 80, 40 women and 40 men), whereas inflammatory variables were measured in a subsample (*n* = 55, 24 women and 31 men). SD: standard deviation; *p* for sex comparisons. SMM: skeletal muscle mass; BFM: body fat mass; BMI: body mass index; WC: waist circumference; NC: neck circumference; CHOL: total cholesterol; HDL-C: high-density lipoprotein cholesterol; LDL-C: low-density lipoprotein cholesterol; VLDL-C: very low-density lipoprotein cholesterol; HbA1c: glycated hemoglobin; hs-CRP: high-sensitivity C-reactive protein, IL-10: Interleukin 10, TNF-α: Tumor necrosis factor-alpha, IL-6: Interleukin 6, IL-18: Interleukin 18.

**Table 2:** Bivariate correlations of NC, WC, and BMI with anthropometric, metabolic, and inflammatory parameters.

Variable		NC		WC		BMI	
		r	*p*	r	*p*	r	*p*
Anthropometric	Weight	0.68	<0.001	0.83	<0.001	0.69	<0.001
	SMM	0.79	<0.001	0.78	<0.001	0.31	0.005
	BFM	0.24	0.035	0.50	<0.001	0.87	<0.001
	BMI	0.41	<0.001	0.61	<0.001	---	---
	WC	0.84	<0.001	---	---	0.60	<0.001
	NC	---	---	0.84	<0.001	0.40	<0.001
Metabolic	CHOL	0.05	0.638	0.01	0.925	−0.27	0.015
	Triglycerides	0.43	<0.001	0.30	0.006	−0.036	0.750
	HDL-C	−0.44	<0.001	−0.40	<0.001	−0.20	0.077
	LDL-C	−0.01	0.877	0.01	0.979	−0.20	0.078
	VLDL-C	0.43	<0.001	0.30	0.006	−0.04	0.753
	Glucose	0.17	0.110	0.19	0.097	0.28	0.013
	HbA1c	0.24	0.03	0.23	0.039	0.13	0.257
	Insulin	0.41	0.004	0.36	0.008	0.22	0.105
Inflammatory	hs-CRP	0.047	0.796	0.11	0.533	0.57	0.001
	TNF-α	0.06	0.743	0.02	0.895	0.04	0.829
	IL-6	0.06	0.711	0.04	0.828	0.16	0.338
	IL-10	0.13	0.440	0.04	0.796	−0.04	0.827
	IL-18	0.05	0.779	−0.04	0.794	−0.08	0.633
	IL-10/TNF-α ratio	0.073	0.680	0.037	0.833	−0.063	0.722

Bivariate correlation coefficients (r) were calculated using Pearson (for anthropometric and metabolic variables) and Spearman (for inflammatory variables) tests according to the results of the normality tests. Anthropometric and metabolic variables were analyzed in the total sample (*n* = 80), whereas inflammatory variables were measured in a subsample (*n* = 55). SMM: skeletal muscle mass; BFM: body fat mass; BMI: body mass index; WC: waist circumference; NC: neck circumference; CHOL: total cholesterol; HDL-C: high-density lipoprotein cholesterol; LDL-C: low-density lipoprotein cholesterol; VLDL-C: very low-density lipoprotein cholesterol; HbA1c: glycated hemoglobin; hs-CRP: high-sensitivity C-reactive protein, IL-10: Interleukin 10, TNF-α: Tumor necrosis factor-alpha, IL-6: Interleukin 6, IL-18: Interleukin 18.

**Table 3:** Sex-stratified correlation coefficients between neck circumference, waist circumference and body mass index with anthropometric, metabolic, and inflammatory variables.

Variable		NC				WC				BMI			
		Women		Men		Women		Men		Women		Men	
		r	*p*	r	*p*	r	*p*	r	*p*	r	*p*	r	*p*
Anthropometric	Weight	0.37	0.019	0.49	0.001	0.58	<0.001	0.84	<0.001	0.69	<0.001	0.90	0.001
	SMM	0.34	0.032	0.40	0.0	0.47	0.002	0.60	<0.001	0.33	0.004	0.50	0.001
	BFM	0.32	0.047	0.44	0.004	0.54	<0.001	0.79	<0.001	0.81	<0.001	0.92	<0.001
	BMI	0.48	0.002	0.52	0.001	0.48	0.002	0.82	<0.001	---	---	---	---
	WC	0.70	<0.001	0.65	0.01	---	---	---	---	0.66	<0.001	0.82	<0.001
	NC	---	---	---	---	0.70	<0.001	0.65	0.017	0.52	<0.001	0.52	0.001
Metabolic	CHOL	0.32	0.043	−0.49	0.001	0.05	0.771	−0.34	0.032	−0.01	0.980	−0.51	0.001
	Triglycerides	0.63	<0.001	−0.04	0.791	0.36	0.020	−0.18	0.263	0.05	0.775	−0.22	0.166
	HDL-C	−0.07	0.678	−0.37	0.020	−0.18	0.276	−0.26	0.101	−0.02	0.928	−0.28	0.085
	LDL-C	0.10	0.535	−0.40	0.010	−0.07	0.686	−0.172	0.290	−0.04	0.804	−0.33	0.034
	VLDL-C	0.64	<0.001	−0.04	0.781	0.36	0.021	−0.18	0.262	0.05	0.760	−0.22	0.163
	Glucose	0.23	0.159	0.04	0.810	0.17	0.293	−0.17	0.296	0.37	0.02	−0.09	0.585
	HbA1c	0.27	0.092	0.05	0.740	0.37	0.018	−0.10	0.562	0.16	0.333	−0.15	0.364
	Insulin	0.31	0.134	0.06	0.725	0.30	0.140	0.16	0.399	0.33	0.112	0.11	0.569
Inflammatory	hs-CRP	0.09	0.745	0.03	0.898	−0.08	0.792	0.21	0.363	0.62	0.008	0.35	0.128
	TNF-α	−0.19	0.526	0.29	0.188	−0.15	0.615	0.13	0.571	−0.12	0.675	0.19	0.423
	IL-6	−0.07	0.824	0.27	0.219	0.24	0.405	−0.03	0.880	0.12	0.673	0.01	0.959
	IL-10	0.77	<0.001	−0.21	0.343	0.46	0.084	−0.24	0.272	−0.07	0.819	−0.06	0.774
	IL-18	0.04	0.866	0.31	0.239	−0.20	0.380	0.29	0.274	−0.10	0.664	−0.01	0.985
	IL-10/TNF-α ratio	0.677	0.008	−0.400	0.081	0.48	0.080	−0.34	0.140	0.15	0.601	−0.29	0.211

Bivariate correlation coefficients (r) were calculated using Pearson (for anthropometric and metabolic variables) and Spearman (for inflammatory variables) tests according to the results of the normality tests. Anthropometric and metabolic variables were analyzed in the overall sample (*n* = 80, 40 women and 40 men), whereas inflammatory variables were measured in a subsample (*n* = 55, 24 women and 31 men). SMM: skeletal muscle mass; BFM: body fat mass; BMI: body mass index; WC: waist circumference; NC: neck circumference; CHOL: total cholesterol; HDL-C: high-density lipoprotein cholesterol; LDL-C: low-density lipoprotein cholesterol; VLDL-C: very low-density lipoprotein cholesterol; HbA1c: glycated hemoglobin; hs-CRP: high-sensitivity C-reactive protein, IL-10: Interleukin 10, TNF-α: Tumor necrosis factor-alpha, IL-6: Interleukin 6, IL-18: Interleukin 18.

**Table 4:** Sex-stratified multiple linear regression models of the association between NC, WC, and BMI with selected metabolic and inflammatory markers, adjusted for age and BMI.

Variable	Women											
	NC				WC				BMI			
	*β*	CI 95%	*p*	*R* ^2^	*β*	CI 95%	*p*	*R* ^2^	*β*	CI 95%	*p*	***R*** **^2^**
CHOL	0.440	0.831, 10.299	0.023	0.071	0.081	−1.388, 2.004	0.715	0.071	−0.02	−3.316, 2.943	0.904	0.05
Triglycerides	0.780	13.745, 28.549	<0.001	0.506	0.560	1.499, 7.631	0.005	0.238	−0.017	−6.663, 5.981	0.914	0.071
HDL-C	−0.023	−2.187, 1.944	0.906	0.022	−0.273	−1.105, 0.242	0.203	0.023	0.029	−1.156, 1.382	0.858	0.005
LDL-C	0.158	−2.168, 4.992	0.429	0.058	−0.075	−1.406, 1.000	0.734	0.074	−0.052	−2.563, 1.875	0.755	0.048
VLDL-C	0.782	2.759, 5.720	<0.001	0.505	0.554	0.288, 1.519	0.005	0.147	0.013	−1.318, 1.214	0.934	0.068
hs-CRP	−0.286	−1.946, 0.559	0.259	0.328	−0.365	−0.810, 0.210	0.227	0.336	0.583	0.168, 1.889	0.023	0.307
IL-10	0.890	0.895, 2.706	0.001	0.569	0.902	0.200, 1.260	0.011	0.356	−0.192	−1.347, 0.751	0.548	0.084
IL-10/TNF-α ratio	0.861	4.406, 8.926	0.005	0.446	0.729	−0.390, 8.461	0.070	0.106	0.187	−5.652, 9.578	0.582	0.148
	**Men**											
CHOL	−0.429	−1.015, −0.812	0.013	0.340	0.186	−1.195, 2.337	0.516	0.225	−0.499	−5.182, −1.416	0.00	0.236
Triglycerides	0.112	−9.225, 16.079	0.586	0.021	−0.001	−5.832, 5.822	0.999	0.029	−0.223	−10.389, 1.962	0.175	0.001
HDL-C	−0.465	−2.576, −0.262	0.018	0.147	−0.193	−0.741, 0.403	0.553	0.010	−0.272	−1.124, 0.094	0.095	0.027
LDL-C	−0.439	−7.509, −0.645	0.021	0.187	0.272	−0.955, 2.403	0.388	0.007	−0.322	−3.652, −0.057	0.044	0.081
VLDL-C	0.109	−1.860, 3.193	0.596	0.020	0.002	−1.160, 1.167	0.995	0.029	−0.224	−2.079, 0.386	0.172	0.001
hs-CRP	0.221	−0.280, 0.587	0.464	0.171	0.130	−0.186, 0.234	0.813	0.144	0.317	−0.049, 0.310	0.144	0.192
IL-10	−0.193	−0.929, 0.515	0.554	0.097	−0.610	−0.496, 0.129	0.233	0.032	−0.091	−0.367, 0.249	0.692	0.060
IL-10/TNF-α ratio	−0.237	−3.308, 1.488	0.433	0.168	0.262	−0.805, 1.368	0.590	0.150	−0.324	−1.785, 0.266	0.137	0.271

Multiple linear regression models were performed using the enter method. Models for neck circumference (NC) and waist circumference (WC) were adjusted for age and body mass index (BMI). Models for body mass index (BMI) were adjusted for age only. Data show standardized regression coefficients (β), 95% confidence intervals (CI), and adjusted *R*^2^. Anthropometric and metabolic variables were analyzed in the full sample (*n* = 80, 40 women and 40 men), whereas inflammatory markers were measured in a subsample (*n* = 55, 24 women and 31 men). CHOL: total cholesterol; HDL-C: high-density lipoprotein cholesterol; LDL-C: low-density lipoprotein cholesterol; VLDL-C: very low-density lipoprotein cholesterol; hs-CRP: high-sensitivity C-reactive protein; IL-10: Interleukin 10; TNF-α: Tumor necrosis factor-alpha.

## Data Availability

The original contributions presented in this study are included in the article. Further inquiries can be directed to the corresponding author.

## Abbreviations

The following abbreviations are used in this manuscript:

BMI	Body mass index
WC	Waist circumference
NC	Neck circumference
TNF-α	Tumor necrosis factor-alpha
IL-6	Interleukin-6
IL-18	Interleukin-18
IL-10	Interleukin-10
EPICO	Estudio para la Pro-resolución de la Inflamación Crónica en Obesidad
INNUGET	Instituto de Nutrigenética y Nutrigenómica Traslacional
ATP III	Adult Treatment Panel III
ISAK	International Advancement of Kinanthropometry
NHANES	National Health and Nutrition Examination Survey
RPM	Revolutions per minute
HDL-C	High-density lipoprotein cholesterol
LDL-C	Low-density lipoprotein cholesterol
VLDL-C	Very low-density lipoprotein cholesterol
HbA1c	Glycated hemoglobin
IL-10/TNF-α ratio	Interleukin-10/Tumor necrosis factor-alpha ratio
hs-CRP	High-sensitivity C-reactive protein
SMM	Skeletal muscle mass
BFM	Body fat mass
CHOL	Total cholesterol
